# Urocortin 3 activates AMPK and AKT pathways and enhances glucose disposal in rat skeletal muscle

**DOI:** 10.1530/JOE-14-0181

**Published:** 2014-11

**Authors:** Manon M Roustit, Joan M Vaughan, Pauline M Jamieson, Mark E Cleasby

**Affiliations:** 1 Department of Comparative Biomedical Sciences, Royal Veterinary College, University of London, Royal College Street, London, NW1 0TU, UK; 1 Laboratory of Neuronal Structure and Function Salk, Institute for Biological Studies, 10010 North Torrey Pines Road, La Jolla, California, 92037, USA; 2 Queen's Medical Research Institute, Centre for Cardiovascular Science University of Edinburgh, 47 Little France Crescent, Edinburgh, EH16 4TJ, UK

**Keywords:** skeletal muscle, urocortin 3, glucose disposal, GLUT4, AMPK, PI3K signalling

## Abstract

Insulin resistance (IR) in skeletal muscle is an important component of both type 2 diabetes and the syndrome of sarcopaenic obesity, for which there are no effective therapies. Urocortins (UCNs) are not only well established as neuropeptides but also have their roles in metabolism in peripheral tissues. We have shown recently that global overexpression of *UCN3* resulted in muscular hypertrophy and resistance to the adverse metabolic effects of a high-fat diet. Herein, we aimed to establish whether short-term local *UCN3* expression could enhance glucose disposal and insulin signalling in skeletal muscle. UCN3 was found to be expressed in right tibialis cranialis and extensor digitorum longus muscles of rats by *in vivo* electrotransfer and the effects studied vs the contralateral muscles after 1 week. No increase in muscle mass was detected, but test muscles showed 19% larger muscle fibre diameter (*P*=0.030), associated with increased IGF1 and *IGF1* receptor mRNA and increased SER256 phosphorylation of forkhead transcription factor. Glucose clearance into the test muscles after an intraperitoneal glucose load was increased by 23% (*P*=0.018) per unit mass, associated with increased GLUT1 (34% increase; *P*=0.026) and GLUT4 (48% increase; *P*=0.0009) proteins, and significantly increased phosphorylation of insulin receptor substrate-1, AKT, AKT substrate of 160 kDa, glycogen synthase kinase-3β, AMP-activated protein kinase and its substrate acetyl coA carboxylase. Thus, UCN3 expression enhances glucose disposal and signalling in muscle by an autocrine/paracrine mechanism that is separate from its pro-hypertrophic effects, implying that such a manipulation may have promised for the treatment of IR syndromes including sarcopaenic obesity.

## Introduction

Impaired insulin-stimulated glucose disposal into skeletal muscle is a major component of the insulin resistance (IR) that develops in advance of type 2 diabetes (T2D) (DeFronzo & Tripathy 2009). In addition, obesity and IR commonly also co-exist with muscular atrophy in the elderly in the syndrome of sarcopaenic obesity (Stenholm *et al*. 2008, Narici & Maffulli 2010, Bassil & Gougeon 2013). Despite this affecting between 4 and 12% of the elderly population (Stenholm *et al*. 2008) and there being several identified common pathways involved in the regulation of muscle size and insulin action (Rommel *et al*. 2001, Sandri *et al*. 2006, Cleasby *et al*. 2007, 2014, Lantier *et al*. 2010), there are no effective treatments available. However, recent work has demonstrated that generalised overexpression of urocortin 3 (UCN3) in mice results in both hypertrophy and increased glucose disposal into muscle (Jamieson *et al*. 2011), making this an interesting candidate for further study.

The UCNs comprise three neuropeptides (UCN1, UCN2 and UCN3) with homology to corticotropin-releasing factor (CRF) that are ligands for CRF receptors (CRFR1 and R2) (Vaughan *et al*. 1995, Hsu & Hsueh 2001, Lewis *et al*. 2001, Reyes *et al*. 2001). CRFR1 is well established as the stress-coping receptor in brain regions, modifying both physiological and behavioural functions. However, CRFR2 and its specific ligands UCN2 and UCN3, in addition to expression in specific brain regions, are also expressed in discrete non-neural tissues in which direct metabolic effects might be expected (Hsu & Hsueh 2001, Lewis *et al*. 2001, Reyes *et al*. 2001).

CRFR2-knockout mice are resistant to high-fat diet (HFD)-induced fat accretion and IR, despite unaltered body weight and increased appetite (Bale *et al*. 2003), apparently due to increased brown fat thermogenesis (Carlin *et al*. 2006). Some of these effects are certainly centrally mediated, as CRFR2 knockdown in the ventromedial hypothalamus (VMH) reduced adipose tissue lipolysis and lipid oxidation (Chao *et al*. 2012). However, UCN2 is expressed in brown adipose tissue and heart of mice and demonstrates autocrine/paracrine cardioprotective effects mediated via ERK1/2, AKT and PKCϵ activation (Brar *et al*. 2002, 2004, Lawrence *et al*. 2005). UCN2 and CRFR2 are both expressed in mouse skeletal muscle (Chen *et al*. 2004, Keipert *et al*. 2013), where they inhibit atrophy and promote hypertrophy (Hinkle *et al*. 2003, Chanalaris *et al*. 2005, Reutenauer-Patte *et al*. 2012). UCN2-knockout mice show increased whole-body insulin sensitivity and resist the effects of an HFD, due to CRFR2-mediated activation of AKT and ERK1/2 signalling in skeletal muscle (Chen *et al*. 2006). Furthermore, CRF stimulates muscle substrate oxidation through the activation of both phosphoinositol 3-kinase (PI3K) and AMP-activated protein kinase (AMPK) pathways (Solinas *et al*. 2006).

Investigations into the role of UCN3 in metabolism are less advanced. Injection of UCN3 into the VMH elevated blood glucose and insulin levels and reduced food intake (Chen *et al*. 2010), while overexpression of UCN3 in the rostral perifornical area of the brain caused increased energy expenditure but a reduction in insulin sensitivity (Kuperman *et al*. 2010). The peripheral effects of UCN3 are clearly significant, however, as global UCN3 knockout increased food intake and reduced insulin sensitivity, while not affecting energy expenditure (Chao *et al*. 2012). Importantly, UCN3 and CRFRs are expressed in pancreatic β cells, where they facilitate insulin secretion in response to high-glucose concentrations (Li *et al*. 2003, 2007). However, although UCN3 is not normally expressed in skeletal muscle, transgenic global UCN3-overexpressing mice showed high levels of UCN3 expression in skeletal muscle, associated with muscle hypertrophy, elevated muscle insulin-like growth factor 1 (IGF1), reduced plasma glucose, improved glucose tolerance and increased glucose disposal into muscle (Jamieson *et al*. 2011). This occurred in the absence of any effect on whole-body insulin sensitivity, although plasma insulin levels and phosphorylation of insulin signalling intermediates in muscle were reduced.

Thus it is likely that CRFR2-mediated effects of UCN3 on metabolism are exerted through distinct central and peripheral actions. Given the positive effects of UCN2 action in skeletal muscle on glucose homeostasis and atrophy resistance and the analogous results generated by whole-body UCN3 overexpression, we aimed to establish whether there might be a paracrine role for UCN3 to improve glucose disposal in skeletal muscle *in vivo* after forced expression. To this end, local overexpression of UCN3 was carried out in a single muscle group and the effects compared with the contralateral control muscles after just 1 week, to enable assessment of the acute tissue-specific effects.

## Materials and methods

### Materials

Molecular reagents were supplied by Promega Corp. and general reagents by Sigma–Aldrich. Antibodies targeting pY608-IRS1, total IRS1, AS160, GLUT1 and total glycogen synthase kinase (GSK) 3α/β were purchased from Millipore (Billerica, MA, USA), β-actin antibody from Sigma and all others from Cell Signaling Technology (Danvers, MA, USA).

### Construction of UCN3 expression vector

pCR–TOPOII containing the full-length mouse UCN3 cDNA (Lewis *et al*. 2001) was consecutively digested with EcoRI, the cDNA insert agarose gel-separated, extracted using a QIAquick gel extraction kit (Qiagen) and phosphorylated using polynucleotide kinase. The *mUcn3* cDNA was then ligated into the dephosphorylated EcoRI-linearised pCAGGS expression vector (Patel *et al*. 2012). The product was used to transform competent JM109 *Escherichia coli*, and correct insertion of cDNA into clones was verified by HindIII digestion and sequencing of minipreps derived from colonies.

### Animals and *in vivo* electrotransfer

All experimental procedures were approved by the Royal Veterinary College's Ethics and Welfare committee and were carried out under UK Home Office licence to comply with the Animals (Scientific Procedures) Act 1986. Male Wistar rats were obtained from Charles River (Margate, UK) at 150–175 g and maintained at 22±0.5 °C under a 12 h light:12 h darkness cycle on a standard chow diet and acclimatised to their new surroundings for 1 week. Preparation and injection of DNA, i.m. injection of hyaluronidase and *in vivo* electrotransfer (IVE) of tibialis cranialis (TC) and extensor digitorum longus (EDL) muscles were carried out under isofluorane anaesthesia as described previously (Cleasby *et al*. 2005, Patel *et al*. 2012). Right TCMs were injected with pCAGGS–mUCN3 and left TCMs with empty pCAGGS vector as within-animal control. The rats were killed by pentobarbitone injection for 1 week later and their muscles rapidly dissected and weighed. The portions of each muscle were fixed in 10% buffered formalin for 48 h and stored in 70% ethanol, snap-frozen in liquid nitrogen-cooled isopentane surrounded in OCT compound (Sakura Finetech, Alphen aan den Rijn, The Netherlands) or freeze-clamped and stored at −80 °C. A total of 26 rats were used.

### Glucose uptake into muscle

Half of the rats were starved overnight, and glucose uptake into paired TC muscles was measured using an intraperitoneal glucose tolerance test (IPGTT), combined with administration of 2-[1,2-^3^H(N)]-deoxy-d-glucose (^3^H-2DG; Perkin-Elmer, Seer Green, Bucks, UK) tracer (Crosson *et al*. 2003, Cleasby *et al*. 2007, 2014). Briefly, ∼5 MBq ^3^H-2DG in 2 mg/kg glucose was administered i.p. and blood samples were collected for the measurement of glucose concentration (Accu-chek Aviva glucometer, Roche Diagnostics) and radioactivity immediately beforehand and 15, 30, 60 and 90 min afterwards. Plasma was separated, deproteinised and counted in Ultima Gold scintillation fluid (Perkin-Elmer) on a beta counter (LS6500, Beckman Coulter, High Wycombe, UK). The powdered muscle was homogenised in dH_2_O, ^3^H-2DG-6-phosphate separated by passage through columns containing AG 1-X8 resin (Bio-Rad) and similarly counted. Tissue glucose uptake was estimated by dividing the ^3^H-2DG-6-phosphate counts by the plasma glucose-specific activity over 90 min and was stated per unit muscle mass.

### Muscle glycogen content

Glycogen was extracted from muscles and quantified as described previously (Chan & Exton 1976). Briefly, TC muscle tissue was digested in 1 M KOH and glycogen precipitated using Na_2_SO_4_ and ethanol. The glycogen pellet was digested overnight at 37 °C using 0.3 mg/ml amyloglucosidase in 0.25 M acetate buffer of pH 4.75. Glycogen content was estimated as the quantity of glucose detected at 490 nm in samples incubated in 0.12 M phosphate buffer of pH 7.0 containing 0.5 mg/ml 4-aminoantipyrine, 1.6 U/ml peroxidase and 10 U/ml glucose oxidase for 25 min at 37 °C, vs a standard curve.

### Determination of muscle fibre size and type distribution

The muscle fibre size was estimated in a blinded fashion in transverse test and control muscle sections of TC mid-belly that were immunostained for laminin using a method adapted from that described previously (Cleasby *et al*. 2007). Fixed tissue was paraffin wax-embedded and 10 μm sections were cut, dewaxed, rehydrated and then antigen retrieval was carried out using 10× Tris–EDTA buffer (pH: 9.0) at 95 °C for 10 min. The sections were blocked for 30 min in blocking buffer (1× PBS, 0.5% Tween 20, 10% goat serum) and incubated overnight with 1:200 rabbit anti-laminin antibody (Sigma), followed by washing 3×10 min in 1× PBS/ 0.5% Tween-20, incubation for 1 h with 1:1000 Alexa Fluor 488 goat anti-rabbit IgG (Invitrogen) secondary antibody and a further 3×10 min washes. The sections were hydro-mounted and images captured using a DM4000B upright microscope and Application Suite software (Leica, Wetzler, Germany). Minimum Feret diameter was measured using Leica QWin software for ∼600 fibres per section and a mean value calculated for each muscle.

Fibre type distribution was determined by simultaneous immunostaining of myosin heavy-chain isoforms (MHC) type I, IIa and IIb of 10 μm TC mid-muscle belly cryosections as described previously (Cleasby *et al*. 2014) using primary antibodies that were a kind gift from Dr Keith Foster, University of Reading, UK. The primary antibodies were visualised using 1:200 dilutions of Alexa Fluor 488, 568 and 633 (Invitrogen) secondary antibodies and the above microscope. The percentage of each fibre type per section was calculated from counts of total numbers of fibres (mean ∼1200 per muscle) and counts of those immunoreactive for MHC I, IIa and IIb, with the percentage of IIx fibres obtained by difference.

### Real-time PCR analysis

Extraction of RNA, preparation of cDNA and relative quantitation of mRNA transcript levels corresponding to most genes of interest was carried out by real-time PCR assay using SYBR Green chemistry (Patel *et al*. 2012) and primers and conditions described previously (Cleasby *et al*. 2014). RNA concentration and quality were assessed using a Nanodrop 1000 (Wilmington, DE, USA) and by visualisation of ribosomal bands after agarose gel electrophoresis. As no SYBR assay could be successfully developed to quantify total mouse and rat UCN3 expression, a mouse UCN3 Taqman assay (Applied Biosystems Mm00453206-s1) was performed to demonstrate relative expression of mUCN3 in muscles vs a dilution series of pCR–TOPOII–UCN3 plasmid. The results are quoted after normalisation to the geometric mean of the mRNA levels of cyclophilin, 36B4 and 18S, expression of which were unchanged by the treatments (data not shown).

### UCN3 RIA

EDL muscles were acid-extracted and partially purified using octadecyl silica cartridges as described previously (Li *et al*. 2003). The purified samples were lyophilised, resuspended in RIA buffer and assayed at several concentrations. The production of antiserum, iodination and purification of synthetic rat/mouse UCN3 (r/mUcn3) analogue for use as tracer, and r/mUCN3 RIA buffers and procedures were similar to those described in detail for inhibin subunits (Vaughan *et al*. 1989). Briefly, the analogue [Tyr^0^Nle^12^] r/mUCN3 was radiolabelled with ^125^I and purified by HPLC using a 0.1% trifluoroacetic acid-acetonitrile solvent system and a diphenyl column. R/m UCN3 antiserum was raised in rabbit using r/mUCN3 coupled to keyhole limpet haemocyanin via carbodiimide. Rabbit 7255 anti-r/mUCN3 was used at a 1:300 000 final dilution and synthetic r/mUCN3 was used as a standard. The EC_50_ and minimal detectable dose for r/mUCN3 were 20 and 1 pg/tube respectively. Closely related CRF family peptides displayed the following crossreactivities: rUCN 1 and rCRF being <0.01%; mUCN 2, 0.5%. This r/mUCN3 RIA employing rabbit 7255 antiserum showed improved sensitivity over the RIA previously published (Li *et al*. 2003); both assays detect UCN3 peptide in murine brain and pancreas, the highest endogenously expressing tissues, in similar quantities.

### SDS–PAGE and immunoblotting

The muscle tissue was homogenised in RIPA buffer using the Ultra-Turrax, followed by rotation for 90 min at 4 °C and centrifugation for 10 min at 16 000 ***g***, and the protein content of the supernatants was quantified using the bicinchoninic acid method (Pierce Biotechnology, Inc., Rockford, IL, USA) using a BSA standard, normalised to the lowest concentration and denatured in Laemmli buffer for 10 min at 65 °C. The aliquots containing 40–80 μg protein were resolved by SDS–PAGE, electro-transferred and immunoblotted as described previously (Cleasby *et al*. 2007, Patel *et al*. 2012). Specific bands were detected by chemiluminescence (Western Lightning Plus, Perkin-Elmer, Waltham, MA, USA) on Fuji Super RX film (Bedford, UK), scanned and quantified using Image J software (NIH, Bethesda, MD, USA). Equal loading was confirmed by blotting for GAPDH protein.

### Statistical analyses

The data are quoted as mean±s.e.m. Comparisons between treated and control muscles were made using paired Student's *t*-tests, after confirming normality of data sets using the Shapiro–Wilk test. The analyses were conducted using Sigma Plot v11.2.0.5 (Systat Software, Inc., Chicago, IL, USA), with *P*<0.05 regarded as significant.

## Results

### Expression of mUCN3 in rat muscle increases fibre size but does not affect total muscle mass or fibre type distribution after 1 week

**Figure 1:**
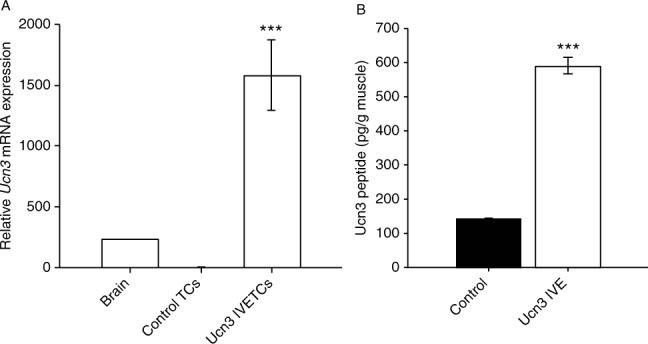
Expression of mUCN3 in rat skeletal muscle. Levels of (A) mouse *Ucn3* mRNA expression in test and paired control tibialis cranialis muscles compared with a whole brain sample, measured using real-time PCR and (B) UCN3 peptide in test and control extensor digitorum longus muscles, measured by RIA, 1 week after IVE. Data are mean±s.e.m. (*n*=8). ***P<0.001 vs paired control.

One week after IVE, expression of mUCN3 was substantially increased at both the RNA (Fig. 1A) and protein (Fig. 1B) levels in test muscles. *mUcn3* RNA was measured in paired TC muscles and was below the limit of detection in left muscles using this assay, consistent with reports that UCN3 is not normally expressed in muscle (Jamieson *et al*. 2011). By way of comparison, test muscle *mUcn3* mRNA expression was several-fold higher than in a positive control sample of mouse whole brain. mUCN3 immunoreactivity was measured in the extracts prepared from whole EDLs and was increased by 4.2-fold in test vs control muscles. Of note, we detected UCN3 peptide in mouse skeletal muscle (Vaughan JM, unpublished observation) in a different highly specific RIA previously described (Li *et al*. 2003) that displays minimal cross-reactivity with UCN2 (<0.01%). Because *Ucn3* mRNA is not normally expressed in muscle, the immunoreactivity observed in EDL may be derived from circulating sources. Nevertheless, it is clear that there is substantially more UCN peptide in the pCAGGS–UCN3 electroporated muscles. Unfortunately, the available antibodies proved unsuitable for immunofluorescence in muscle tissue, and therefore we could not assess the transfection efficiency of UCN3 by this method. However, the RIA data combined with the set of consistent metabolic, gene expression and signalling data presented below are suggestive that physiologically relevant concentrations of UCN3 were achieved.

**Figure 2:**
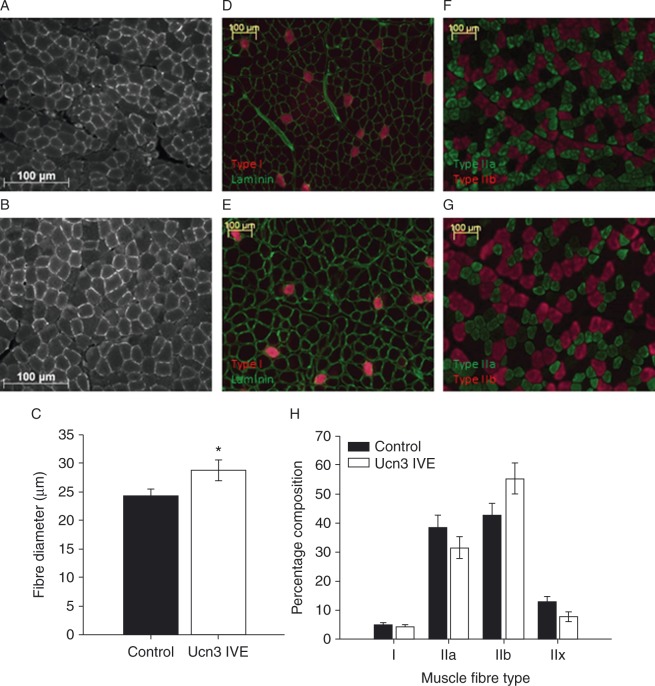
Expression of mUCN3 for 1 week resulted in increased muscle fibre size in the absence of a change in muscle mass, and no change in fibre type distribution. Muscle fibre diameter was measured in fixed transverse sections of TC muscles immunostained for laminin. Representative sections from (A) control and (B) mUCN3-expressing muscles are accompanied by (C) summary data. The percentage of each fibre type present in TC muscles was calculated after immunostaining of frozen transverse sections for type I, IIa and IIb fibres, with type IIx fibres indicated by lack of immunostaining. (D) Control and (E) mUCN3-expressing muscles immunostained for type I myosin heavy chain (MHC; red) and laminin (green). (F) Control and (G) mUCN3-expressing muscles immunstained for type IIa MHC (green) and type IIb MHC (red). (H) Summary fibre type distribution. Scale bars: 100 μm, *n*=6–8. Data are mean±s.e.m. *P<0.05 vs paired control. Black bars, control; white bars, UCN3 IVE muscle.

There was a mean 19% increase in TC fibre diameter over this period in mUCN3-expressing muscles (*P*=0.030; Fig. 2A, B and C), which was not accompanied by a change in the coefficient of variation between test and control muscles (data not shown). However, this short duration of expression was not sufficient to affect the mass of whole TC or EDL muscles (TC: test 0.378±0.018 g, control 0.379±0.014 g; EDL: test 0.133±0.0053 g, control 0.135±0.0045 g; *n*=10). In addition, the percentage of type I, IIa, IIb and IIx fibres comprising each TC muscle was not altered at this time point (Fig. 2D, E, F, G and H; *n*=6–8), although it seemed that there was a tendency for there to be fewer IIx (*P*=0.07) and more IIb (*P*=0.08) fibres in mUCN3-expressing muscles.

### mUCN3 expression is associated with increases in the expression of IGF and its receptor, but also of selected pro-atrophic genes

**Figure 3:**
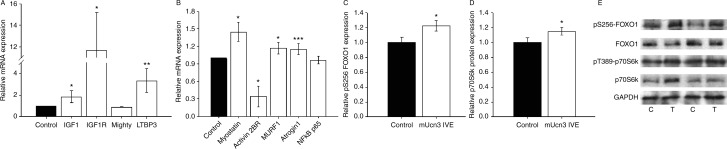
Muscle UCN3 expression has contrasting effects on mediators of muscle hypertrophy and atrophy. Effects of local muscle UCN3 expression on relative mRNA and protein expression and phosphorylation of key mediators in pathways regulating muscle mass. Expression data were obtained by real-time PCR analysis of mRNA extracted from mUCN3-expressing and control TC muscles and are shown normalised to control. (A) mRNA levels of pro-hypertrophic genes: *IGF1*, IGF1 receptor (*IGF1R*), mighty (akirin1) and latent transforming growth factor-β 3 (*LTBP3*). (B) mRNA levels of pro-atrophic genes: myostatin, activin IIB receptor (activin 2BR), muscle ring finger protein 1 (*MURF1*), atrogin1 and the p65 subunit of nuclear factor κB (*NF* *κ* *B*–*p65*). (C) *pS256*–*FOXO1* and (D) p70S6k total protein levels and (E) sample immunoblots are shown. Total FOXO1 protein and pT389–p70S6k were not affected by forced UCN3 expression. C, control; T, test. Data are mean±s.e.m.; *n*=8; *P<0.05, **P<0.01, ***P<0.001 vs paired control.

In order to establish whether particular pro-hypertrophic or pro-atrophic pathways were activated at this early time point of local UCN3 expression in TC muscle, potentially explaining the modest increase in muscle fibre size observed, expression and activation of key mediators in these pathways were assessed using real-time PCR and immunoblotting. Firstly, consistent with the effects of transgenic overexpression (Jamieson *et al*. 2011), mRNA expression of both *IGF1* and *IGF1R* were upregulated in UCN3 OE muscles (by 87 and 1067%, *P*=0.049 and *P*=0.014 respectively; Fig. 3A). Secondly, pS256–FOXO1 was increased by the manipulation (by 22%, *P*=0.032; Fig. 3C/E), implying the inhibition of its transcriptional activity. However, although increased FOXO1 activity would be expected to suppress the expression of pro-atrophic E3 ubiquitin ligases (Stitt *et al*. 2004), in fact mRNA levels of both MURF1 and Atrogin1 were slightly increased in these muscles (by 17 and 15%, *P*=0.049 and *P*<0.001 respectively; Fig. 3B).

mRNA expression of the pro-atrophic transforming growth factor-β family member myostatin was in fact increased (by 45%, *P*=0.030; Fig. 3B). However, this effect would be unlikely to limit increases in muscle size, as expression of its receptor, the activin 2B receptor, was reduced by 60% (*P*=0.042; Fig. 3B; Lee & McPherron 2001, Cleasby *et al*. 2014). Furthermore, mRNA expression of latent transforming growth factor β-binding protein-3 (LTBP3), which impairs myostatin signalling (Anderson *et al*. 2008), was increased (by 233%, *P*=0.010; Fig. 3A). Finally, no change in mighty (akirin-1) expression, a downstream target of myostatin in muscle (Marshall *et al*. 2008), was detected (Fig. 3A).

Although there was a small increase in total p70S6k protein (by 15%, *P*=0.011), no change in pT389–p70S6k was shown (Fig. 3D/E), suggesting that this kinase was not involved in the phenotype. In addition, mRNA expression of the pro-atrophic *NF* *κ* *B* *–* *p65* subunit was also unaffected by forced UCN3 expression (Fig. 3B).

### mUCN3 expression enhances glucose uptake into muscle during an IPGTT and increases cellular glucose transporter content

**Figure 4:**
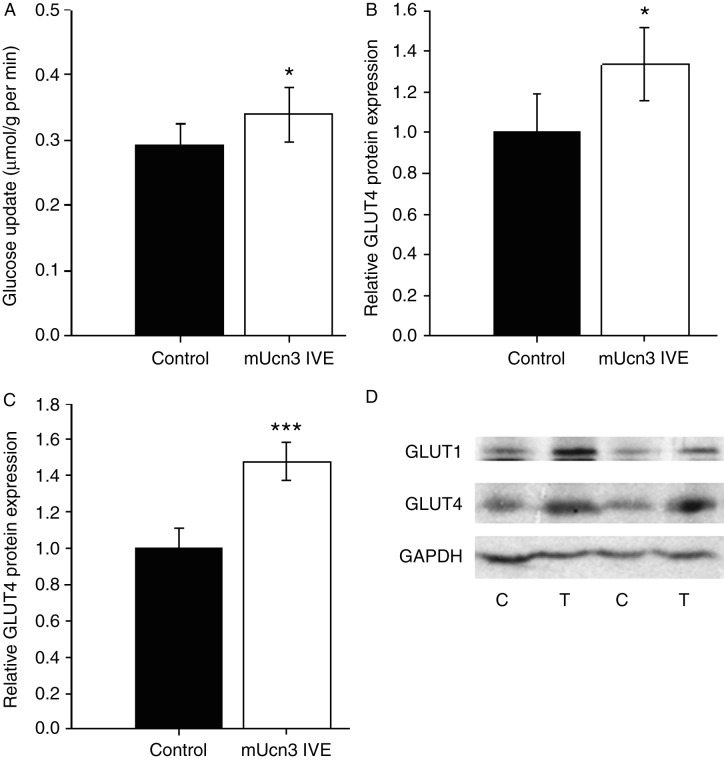
mUCN3 expression enhances muscle glucose disposal and increases protein levels of GLUT1 and GLUT4 glucose transporters. (A) Glucose uptake into paired TC muscles, estimated using 2-[1,2-^3^H(N)]-deoxy-d-glucose tracer during an intraperitoneal glucose tolerance test. Summary data for (B) GLUT1 and (C) GLUT4 protein contents quantified by western immunoblotting of mUcn3 expressing and paired control muscle lysates, normalised to control. (D) Sample immunoblots for GLUT1, GLUT4 and GAPDH, which was unaltered by the manipulation. Data are mean±s.e.m., *n*=8–9. *P<0.05, ***P<0.001 vs paired control.

Glucose uptake into mUCN3 expressing and paired control TC muscles were also assessed after 1 week. The IPGTTs carried out in these rats resulted in typical plasma glucose excursions (data not shown). Despite the lack of effect on gross muscle mass, glucose uptake into test muscles was enhanced (by 23%, *P*=0.032; Fig. 4A) on a per unit mass basis, implying that UCN3 mediates enhanced muscle glucose disposal by a mechanism that is not totally dependent on hypertrophy. The enhanced glucose disposal was associated with increases in total cell levels of both the GLUT1 (by a mean 34%, *P*=0.026; Fig. 4B/D) and GLUT4 (by a mean 48%, *P*=0.0009; Fig. 4C/D) glucose transporter proteins. The associated increased capacity for insulin-stimulated glucose uptake likely contributed to the increased clearance observed and these data suggest that basal glucose uptake may also be increased by muscle UCN3 expression. However, glycogen storage by the muscles at the 1 week time point was unaffected (data not shown), suggesting that the additional glucose taken up is being utilised rather than stored.

### mUCN3 expression increases both phosphorylation and total protein expression of PI3-kinase pathway signalling intermediates

**Figure 5:**
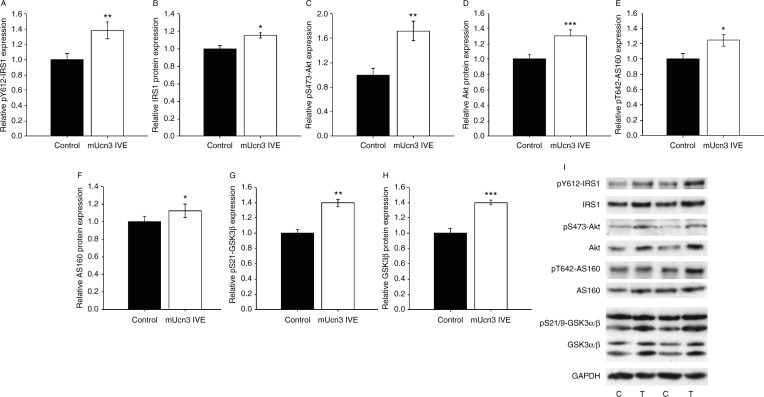
Enhanced muscle glucose disposal is associated with increased phosphorylation and protein levels of phosphoinositol 3-kinase pathway intermediates. Western immunoblotting for phosphorylation of regulatory residues and total protein content of PI3-kinase pathway intermediates was undertaken using mUCN3 expressing and paired control TC muscle lysates. Summary data for (A) pY612-IRS1, (B) total IRS1, (C) pS473–AKT, (D) total AKT, (E) pT642-AS160, (F) total AS160, (G) pS21–GSK3β and (H) total GSK3β are shown. (I) Sample immunoblots for each protein target and GAPDH, levels of which were unchanged by the manipulation, as were pS21–GSK3α and total GSK3α. Data are mean±s.e.m., *n*=8. *P<0.05, **P<0.01, ***P<0.001 vs paired control.

To assess whether muscle mUCN3 expression might also impact glucose disposal through increased activation of the PI3-kinase signalling pathway, protein expression levels and phosphorylation of intermediates at regulatory residues were assessed in lysates generated from paired TC muscles removed from fed, otherwise untreated rats. Interestingly, significant increases were detected in both phosphorylation and total protein levels of all the intermediates assessed in UCN3-electroporated muscles, in the absence of any effect on either GAPDH or β-actin levels. Specifically, pY612-IRS1 and total IRS1 were increased by 38 and 15% respectively (*P*=0.008 and *P*=0.024; Fig. 5A/B/I), pS473–AKT and total AKT were increased by 72 and 30% (*P*=0.005 and *P*=0.0003; Fig. 5C/D/I), pT642–AKT substrate of 160 kDa (AS160) and total AS160 by 24 and 12% (*P*=0.026 and *P*=0.047; Fig. 5E/F/I) and pS9–GSK3β and GSK3β both by 40% (*P*=0.0011 and *P*=0.0002; Fig. 5G/H/I). Phosphorylation and protein levels of GSK3α were unaltered (Fig. 5I). Thus the UCN3-induced increase in glucose disposal may also be contributed to by increased flux through the PI3-kinase pathway from at least the level of IRS1, likely mediated by the increased IGF1 binding to its receptor (Fig. 3A; Rommel *et al*. 2001, Jamieson *et al*. 2011).

### mUCN3 expression also activates AMPK in muscle

**Figure 6:**
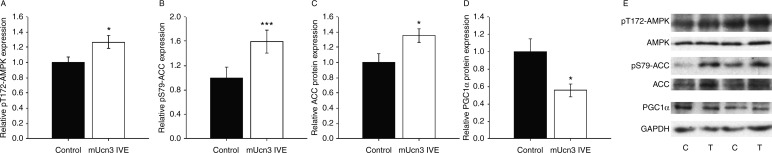
Muscle mUCN3 expression increases activation of AMPK but decreases PGC1α. Western immunoblotting for (A) pT172–AMPK, (B) pS79–ACC, (C) total ACC and (D) total PGC1α protein was undertaken using mUCN3 expressing and paired control TC muscle lysates. Summary data and (E) sample immunoblots for each protein target and the loading control GAPDH are shown. Levels of total AMPK and GAPDH proteins were unchanged by the manipulation. Data are mean±s.e.m., *n*=8. *P<0.05, ***P<0.001 vs paired control.

As muscle glucose uptake is also mediated by activation of AMPK, phosphorylation and protein expression of AMPK and its substrate acetyl coA carboxylase (ACC) were quantified by western blotting analysis. UCN3 expression resulted in a 27% increase in pT172–AMPK (*P*=0.024; Fig. 6A/E), implying increased activation, accompanied by a consistent 59% increase in pS79–ACC (*P*=0.0001; Fig. 6B/E) and also an increase in total ACC protein (by 36%, *P*=0.023; Fig. 6C/E). Total AMPK protein remained unchanged. However, surprisingly, PGC1α levels were reduced (by 44%, *P*=0.0020; Fig. 6D/E) implying that the AMPK activation would be unlikely to result in increased mitochondrial biogenesis and oxidation (Wu *et al*. 1999, Mootha *et al*. 2003). Nevertheless, our findings imply that multiple changes in signalling molecules that promote glucose uptake arise as a result of UCN3 expression in TC muscle.

## Discussion

In this study, we aimed to establish the effects of a short period of local overexpression of UCN3 on glucose disposal by skeletal muscle to identify the potential molecular mediators of any effect. This manipulation resulted in a modest increase in myofibre diameter in the electroporated muscle, but did not generate a detectable difference in whole-muscle mass after only 1 week, analogous to the dose-dependent effects of UCN2 peptide administration to mice (Hinkle *et al*. 2003). However, UCN3 expression enhanced glucose disposal in treated vs paired control TC muscles on a per unit mass basis, indicating that this effect of UCN3 expression is at least in part exerted through an autocrine/paracrine mechanism and also that it was not an indirect effect of increased muscle mass. This positive effect on glucose disposal occurred after administration of a glucose load and may have been mediated by the activation of the insulin signalling pathway, the concurrent AMPK activation and/or the increased GLUT1/4 protein expression observed.

The 1 week time point was chosen to give the UCN3 construct time for expression and to minimise any inflammation resulting from the IVE procedure (Cleasby *et al*. 2005), while avoiding the longer term effects that gross hypertrophy might have on glucose disposal and the potential confounding effects of the whole-body germ-line manipulation carried out previously (Jamieson *et al*. 2011). Nevertheless, increased muscle fibre diameter was observed and this was associated with elevations in both IGF1 and IGF1R expression, indicating that gross hypertrophy consistent with our previously published work (Jamieson *et al*. 2011), the effects of UCN2 (Hinkle *et al*. 2003, Reutenauer-Patte *et al*. 2012) and the pro-hypertrophic effects of all UCNs in cardiomyocytes (Chanalaris *et al*. 2005), would likely develop after a longer period of forced expression. It is known that IGF1 is sufficient to cause activation of the PI3K–AKT pathway and both increased muscle mass and glucose uptake (Di Cola *et al*. 1997, Rommel *et al*. 2001, Palazzolo *et al*. 2009), but the details of the signalling mechanisms whereby elevated IGF1 might impact upon these parameters was unclear in our previous work (Jamieson *et al*. 2011).

In addition to the observed inconsistency between increased insulin sensitivity in CRFR2 and UCN2-knockout mice (Bale *et al*. 2003, Carlin *et al*. 2006, Chen *et al*. 2006) and increased glucose tolerance in the absence of any effect on insulin sensitivity in UCN3-overexpressing mice (Jamieson *et al*. 2011), localised UCN3 expression in muscle resulted in an increase in glucose uptake associated with changes in signalling consistent with increased muscle insulin sensitivity after a glucose load. These differences are probably related with the universal changes in UCN3 or CRFR2 expression having disparate influences on central and peripheral control mechanisms or feedback effects of pronounced hypertrophy on AKT and/or AMPK signalling. The study described here compared the effects of acute local UCN3 expression in muscle vs a within-animal control and therefore permits a clearer insights into the direct autocrine/paracrine effects of UCN3 in muscle.

The UCN3-mediated increase in glucose uptake was associated with the phosphorylation of a number of intermediates in both the PI3K–AKT–FOXO1 and AMPK signalling pathways, indicating increased activity, both or either of which could mediate this effect. In addition, total protein levels of many of these molecules were also increased, suggesting an effect of UCN3 expression at the level of translation or above. Consistent with this, we observed increased uncoupling protein and *IGF1* mRNA in UCN3 transgenic mice (Jamieson *et al*. 2011), while UCNs have also been shown to activate transcription factors in macrophages (Tsatsanis *et al*. 2006). These data contrast with the reduced IRS1 and AKT phosphorylation observed in the transgenic mice (Jamieson *et al*. 2011), which may have been the result of the reduced circulating insulin levels or a compensatory effect for the chronic global overexpression. In support of a role for both the PI3K and AMPK pathways in mediating the effects of short-term mUCN3 expression in muscle, CRF was shown to cause increased substrate oxidation in muscle that relied on both AMPK and PI3K activation (Solinas *et al*. 2006). In contrast, UCN2 increased glucose uptake in the heart through a mechanism that required AMPK but not AKT activation (Li *et al*. 2013), while the hypertrophic effects required AKT (Chanalaris *et al*. 2005). However, in addition, AMPK activation may have beneficial effects on muscle mass through the inhibition of apoptosis and promotion of normal autophagy (Luo *et al*. 2013). Thus, additional research is still required to assess the importance of each pathway in the phenotype of enhanced glucose disposal and muscular hypertrophy and to establish how they are being activated by UCN3.

Acute UCN3 expression did not alter glycogen storage in the rat muscles, while the increased glucose uptake in global UCN3-overexpressing mice was reflected in increased glycogen storage (Jamieson *et al*. 2011), implying either that utilisation was also increased in the IVE test muscles, or that this is a feature of longer term expression. The observed reduction in PGC1α levels also suggests that UCN3 does not have the mitochondrial preservation effects recorded in cardiac muscle (Kuizon *et al*. 2009). This effect does not seem to be the result of a UCN3-mediated shift towards type II glycolytic fibres, as seen in the transgenic model, as type I fibre percentage was unchanged, although this again may reflect the short timescale of the study. In addition, as the majority of the data presented have been obtained in the predominantly fast twitch TC muscle, it may be that muscles with alternative fibre compositions would demonstrate different results. In addition, we observed increased expression of pro-atrophic pathways in this model, which might seem surprising at first glance, given that activation of AKT and FOXO1 normally switch off such expression (Stitt *et al*. 2004, Latres *et al*. 2005). It may be that this reflects counter-regulatory changes in the activity of an alternative transcription factor, for example FOXO3 (Zheng *et al*. 2010), or that our data corroborate the recently published work that implies a requirement for atrogin as part of the process of remodelling involved in muscular hypertrophy (Baehr *et al*. 2014).

The effect of local UCN3 expression on muscle fibre size and the further enhancement of glucose disposal beyond the level that would be expected purely as a result of the hypertrophy is very similar to the phenotype we observed recently after adeno-associated virus-mediated local inhibition of myostatin action in muscle (Cleasby *et al*. 2014), despite the modest upregulation of myostatin mRNA we observed here, which has also been observed in another model of rat muscle hypertrophy (Abo *et al*. 2012). The effects of myostatin inhibition occurred despite a reduction in IGF1 expression and reduced activating phosphorylation of AMPK and AKT, but as here, increased levels of cellular GLUT1 and GLUT4 proteins resulted, implying increased capacity for both basal and insulin-stimulated glucose uptake. However, we have not as yet established a causal link between these variables, while glucose clearance into muscle is determined not only by the capacity for transporter-mediated facilitated diffusion, but also by other physiological variables, including capillary density, vascular smooth muscle tone and hexokinase activity. Notably, UCN3 has been shown to cause arterial vasodilatation (Venkatasubramanian *et al*. 2013), thus a paracrine effect of mUCN3 on muscle arterioles could also contribute to the observed effect. Nevertheless, targeting of CRFR2 or myostatin in muscle may therefore provide potential for treatment of IR and atrophic syndromes to complement nutrition and exercise-based interventions (Bassil & Gougeon 2013). Although UCN3 is not normally expressed in rodent muscle, it is expressed in human muscle (Hsu & Hsueh 2001), suggesting that interventions targeting this pathway may be of more physiological relevance in this species.

Thus, we have shown that forced expression of UCN3 in skeletal muscle enhances local glucose uptake by an autocrine/paracrine mechanism associated with activation of both PI3-kinase-Akt and AMPK pathways and increased glucose transporter expression. This occurs in addition to a hypertrophic effect, implying that local CRFR2 agonism may be a useful therapeutic approach in the treatment of IR syndromes including T2D and sarcopaenic obesity.

## Author contribution statement

M E C and P M J conceived and designed the experiments. M M R, M E C and J M V collected data. M E C and M M R analysed and interpreted data. M E C and P M J drafted and revised the manuscript.
